# Patella resurfacing during total knee arthroplasty is cost-effective and has lower re-operation rates compared to non-resurfacing

**DOI:** 10.1186/s13018-021-02295-8

**Published:** 2021-03-11

**Authors:** Thomas Parsons, Talal Al-Jabri, Nick D. Clement, Nicola Maffulli, Deiary F. Kader

**Affiliations:** 1 South West London Elective Orthopaedic Centre, Denbies Wing, Epsom General Hospital, Surrey, KT18 7EG UK; 2 Royal United Hospital Bath, Combe Park, Bath, Avon BA1 3NG UK; 3 Department of Surgery and Cancer, Imperial College London, London, SW7 2AZ England; 4 Department of Orthopaedics, Royal Infirmary of Edinburgh, Little France, Edinburgh, EH16 4SA UK; 5 Department of Medicine, Surgery and Dentistry, University of Salerno, Via S. Allende, 84081 Baronissi, SA Italy; 6 Queen Mary University of London, Barts and the London School of Medicine and Dentistry, Centre for Sports and Exercise Medicine, Mile End Hospital, 275 Bancroft Road, London, E1 4DG England; 7 School of Pharmacy and Bioengineering, Keele University School of Medicine, Stoke on Trent, ST5 5BG UK; 8 University of Kurdistan Hewler, Erbil, Iraq

**Keywords:** Arthroplasty, Patella, Knee replacement, Resurfacing

## Abstract

**Background:**

The decision to resurface the patella as part of total knee arthroplasty may be influenced by the surgeon’s preference, education, training, tradition and geographic location. Advocates for non-resurfacing or selectively resurfacing may claim no difference in patient reported outcomes, and that resurfacing is associated with increased risks such as extensor mechanism injury or malalignment, problems with the design of the patella component and technical issues intraoperatively.

**Aims:**

To critically examine factors that should be considered in addition to patient reported outcomes in the decision process of resurfacing or non-resurfacing of the patella in total knee arthroplasty.

**Method:**

A comprehensive literature search was conducted to identify factors that may influence decision making in addition to knee specific patient reported outcome measures such as surgical risks, patient quality of life, procedure cost, re-operation rate, implant design, surgeons learning curve and the fate of remaining cartilage in native patellae.

**Results:**

Patient-reported outcomes are equivocal for resurfacing and non-resurfacing. Critical analysis of the available literature suggests that the complications of resurfacing the patella are historic, which is now lower with improved implant design and surgical technique. Routine resurfacing was cost-effective in the long term (potential saving £104 per case) and has lower rates of revision (absolute risk reduction 4%). Finally, surgical judgment in selective resurfacing was prone to errors.

**Conclusion:**

Patella resurfacing and non-resurfacing had similar patient-reported outcomes. However, patella resurfacing was cost-effective and was associated with a lower rate of re-operation compared to non-resurfacing.

## Background

The practice of patella resurfacing as part of total knee arthroplasty (TKA) remains heterogenous, with rates of primary patella resurfacing (PPR) ranging from 4% in Norway to 90.6% in the United States of America (AJRR 2018 Annual Report) [1]. The Australian Registry has shown an increase in PPR, raising from 41.5% in 2005 to 66.6% in 2017. In contrast, the rate of PPR in Sweden has continued to decrease since the mid 1980’s, and in 2017 was performed in only 2.6% of TKA procedures. According to the 17th Annual Report of the United Kingdom (UK) National Joint Registry (NJR), just over one third of all TKAs registered underwent patella resurfacing [2].

Knee specific patient-reported outcome measures (PROMs) naturally assume priority when assessing whether PPR should be undertaken [3]. However, it is essential to consider other factors such as re-operation rate, patient quality of life, cost-effectiveness, influence of implant design, surgical risks, surgeons’ learning curve and the fate of the remaining patella articular cartilage when not resurfaced.

The current review examined factors that surgeons may want to take into consideration include the following:

1. PROMs of PPR verses primary patella non-resurfacing (PPNR)
2. Re-operation rate and outcomes of secondary patella resurfacing
3. Patient quality of life lost and inconvenience
4. Intra-operative time spent at PPR versus re-admission for secondary patella resurfacing (SPR)
5. The costs of patella resurfacing
6. Influence of implant design on outcomes
7. Surgical risks of primary patella resurfacing (patella fracture, osteonecrosis, aseptic loosening, instability, under or overstuffing, patella clunk syndrome and rupture of the extensor mechanism)
8. The fate of non-resurfaced patellae;
  - Cellular changes of the articular cartilage of the patella when everted during surgery
  - Interaction of metal on cartilage for retained patellae

## Methodology

A review of the literature with a search strategy was undertaken to identify relevant articles in relation to patella resurfacing in TKA. This included a search on MEDLINE and Google Scholar from January 2010 to November 2020. Search terms included (‘total knee arthroplasty’ [All fields] OR ‘knee arthroplasty’ [MeSH terms] with all entry terms and ‘patella’[All fields]) OR (‘resurfacing’ [MeSH terms], OR non-resurfacing’ [MeSH terms]). Journals in all languages were included without any limitations in the search strategy. Abstracts were screened for relevance. Exclusion criteria included letters, editorials and studies identified as being poorly designed and of a low level of evidence. References of selected full-text articles were screened for the inclusion of additional articles. The selected articles were critically appraised by the authors and arguments for and against resurfacing were reviewed.

## Results

### PROMs of PPR vs PPNR

Multiple recent randomised controlled trials (RCTs) and meta-analyses demonstrate comparable clinical outcomes of TKA with and without patella resurfacing [4–9]. The most recent meta-analysis of 20 randomised control trials (RCTs) found that both the knee and functional components of the Knee Society Score (KSS) were statistically significantly, although not clinically significantly, better in those undergoing PPR in additional to a lower re-operation rate for any reason and re-operation for anterior knee pain (AKP) [10]. However, all other outcomes, including AKP, re-operation for non-patellofemoral complications, patient satisfaction, Oxford Knee Score, Knee Injury and Osteoarthritis Outcome Score and range of motion, showed no significant difference.

The only randomised-control trial (RCT) comparing PPR, PPNR and selective patella resurfacing showed significantly higher (better) PROMs for the PPR and selective resurfacing groups compared with the PPNR group at 5 years [11]. Within the selective patella resurfacing group, those who underwent resurfacing had higher PROMs (significance not mentioned).

The analysis of 28 studies in a review by The National Institute for Health and Care Excellence (NICE) achieved similar results, suggesting no difference in terms of PROMs, revision (excluding patella-only revision), length of stay and deep vein thrombosis at multiple follow-up points [12]. The same review also found that PPR was beneficial with respect to quality of life and stiffness in the short term and minor revision, and re-operation in the long term.

### Key points

There is no evidence that PPR or PPNR has a clinically meaningful difference in knee specific patient reported outcomes.

### Re-operation rate and outcomes of secondary patella resurfacing

Ultimately, surgeons may be concerned about the proportion of patients that may require further surgery after TKA (Table 1). Advocates for routine PPR quote a reduced rate of further operations. A 2012 meta-analysis demonstarted rates of patellofemoral complications after TKA with PPR versus PNRR was 5.9% and 12.6%, respectively [14]. In the same analysis, re-operation for patellofemoral joint (PFJ) problems was 1.2% for the PPR group and 3% for the PPNR group. This was corroborated by a meta-analysis of 14 RCTs by Chen et al. that found absolute risk of re-operation was reduced by 4% (95% confidence interval [CI] 2–6%) in the PPR group [6]. Pakos et al. also demonstrated an absolute risk reduction of 4.6% of re-operation for PPR [16].

**Table 1:** Rates of re-operation for patella-femoral problems from meta-analyses

Rate of re-operation for patellofemoral problems		
PPR	PPNR	Reference
2.3%	6.5	Nizard et al. 2005 [13]
−	8.7%	Parvizi et al. 2005 [4]
1.2%	3%	Pilling et al. 2012 [14]
0.8%	4.8%	Johnson et al. 2012 [15]
1%	4.6%	Teel et al. 2019 [10]

*PPR* primary patella resurfacing, *PPNR* primary patella non-resurfacing

A study comparing the outcomes of 10,157 TKAs from a regional arthroplasty registry demonstrated a higher rate of patella-only revision (4.8% vs 0.8%) and all cause revision (8.2% vs 6.3%) for PPNR and PPR respectively [15]. Progressive patellofemoral arthritis was the indication for 87% of patella-only revisions and 53% of all cause revisions in the PPNR group.

A systematic review of registry data from six countries showed disparate practice secondary to geography as previously described and showed lower re-operation rates for PPR [17].

The cumulative revision rate for all unconstrained TKAs in the UK NJR 2020 Annual Report was 4.28% with the patella resurfaced and 5.09% without patella resurfacing at 15 years. The mean age of both groups was 70 years, and in total 435168 resurfaced patellae are registered in the NJR versus 709884 un-resurfaced patellae [2].

A 2019 meta-analysis found re-operation rates for any reason after TKA were 10.6% for PPR and 14.7% for PPNR (risk ratio 0.71 [0.59–0.84]), re-operation rate for anterior knee pain was 1.0% for PPR and 4.6% for PPNR (risk ratio 0.28 [0.17–0.45]) and re-operation for PFJ problems excluding AKP was 2.1% for PPR and 1.2% for PPNR (odds ratio 1.55, 95% CI 0.90 to 2.67]) [10]. The number needed to treat with PPR to prevent 1 further re-operation was 25 [10].

Secondary patella resurfacing (SPR) anecdotally benefits only 50% of patients, while exposing all of the patients them to further surgical and anaesthetic risks [12]. In one of the largest reported series of SPR outcomes using the Trent and Wales Arthroplasty Register, only 44% of patients benefitted from SPR [18]. Furthermore, 13% of patients underwent further surgery on the same knee following SPR, the majority were for persistent knee pain. Parvizi et al. studied the outcomes of 39 patients following SPR: 18% had further surgery for infection, patella maltracking or revision TKA [19].

Selective patella resurfacing has the potential to achieve optimum outcomes by resurfacing those at high risk of anterior knee pain whilst reducing risk of post-operative complications associated with patella resurfacing. In practice, assessment of patient symptoms, pre-operative imaging and intra-operative findings are unreliable predictors of TKA outcomes [11, 20, 21].

### Key points

The re-operation rate following PPNR was 1–8.7%. The outcomes are mixed and difficult to predict. Whether initial PPR in this group of patients would have prevented post-operative pain is not possible to conclude; however, they have undergone two operations, thereby exposing them to additional risk.

### Patient quality of life and inconvenience

An RCT involving 1715 TKAs followed up over 10 years found that PPR was associated with non-significant increased gain is quality of life years (QALYs) (0.187; 95% CI − 0.025 to 0.399) [22]. Data from the Australian joint registry and the NJR also suggests that PPR have a relative QALY gain of 0.64 over PPNR over the patients lifetime [23].

No measure of patient inconvenience was identified in the literature, but greater inconvenience is likely to be experienced by patients after PPNR if they are at higher risk of undergoing further procedures for which they must prepare, undergo and then recover from. A study of SPR outcomes recorded the time between primary TKA and SPR at a median of 28 months (interquartile range 20–42) [24], similar to that reported by Parvizi et al. of 29 months (range 24–92) [19]. Therefore, a proportion of patients will endure approximately 2 years of dysfunction before conversion to SPR.

### Key points

Available evidence suggests PPR provides an increased quality of life advantage.

### Intra-operative time spent at PPR vs time for SPR

An advantage of PPNR is avoidance of an operative step with associated reduced operative time. An RCT involving 116 surgeons at 34 centres recorded an average TKA took 2 hours including anaesthetic room and operating theatre time [22]. PPR increased the average operative time by 3 minutes [22], which is clinically non-relevant. Pilling et al. found no significant difference in operative time [14]. Perhaps more importantly, such a time saving is highly unlikely to allow for an additional patient to be added to the operating list on that day, negating any cumulative gain of time. Furthermore, patella resurfacing does not produce a higher rate of infection [14].

### Key points

There is no evidence patella resurfacing takes significantly longer intra-operatively.

### Economy of patella resurfacing

The 17th annual report of the UK NJR has established that approximately 100,000 TKA are performed annually, with an estimated cost of £550 million. The NJR also identifies that only one third of surgeons resurface the patella routinely [2]. This decision has substantial monetary implications.

A recent report by NICE suggests there is likely a cost-economic benefit from routine patella resurfacing, but does not make this a formal recommendation [12].

Those surgeons in favour of resurfacing argue that anterior knee pain can develop or persist postoperatively if the patella is not resurfaced, and this can result in the need for secondary resurfacing. However, a secondary procedure often presents to family physicians and physiotherapists prior to the orthopaedic surgeon, and this accrues cross-specialty clinic appointments with further investigations and ultimately an elective admission, use of consumables, an operating theatre session and follow-up care all of which have financial repercussions.

Surgeons who oppose resurfacing may argue that resurfacing could prolong operative duration, would cost more (from component costs), and is not without complication. This could subsequently lead to the clinical and economic burden of revision surgery [25, 26]. Furthermore, the importance of patellofemoral joint mechanics has become more established over the last two decades, and this has led to more ‘patella-friendly’ femoral component designs with sided and deeper trochlear grooves. The patella-friendly TKA and the un-resurfaced patellae aim to reduce anterior knee pain and improve patellofemoral tracking. However, these modernised designs have not been successful in restoring normal morphology [27, 28].

To determine whether patella resurfacing is cost-effective, a review of the current literature identified two key studies and was consistent with the 2020 NICE guidelines for patella resurfacing [12, 22, 23]. These are summarised in Table 2.

**Table 2:** Summary of studies evaluating cost-effectiveness of patella resurfacing

Study	Design and methodology	Results	Conclusion
Weeks et al. [23]	Cost-effectiveness analysis using data from the 2014 Australian joint registry and applied to the Canadian market. Quality adjusted life years (QALY) were the utility scores used. Study duration: projected over 14 years. 443,948 patients extracted from the Australian NJR 225,915 patients were not resurfaced and 218,033 were resurfaced.	9240 revisions in the un-resurfaced group vs 5992 revisions in resurfaced group. Mean total treatment cost per patient for un-resurfaced vs resurfaced patellae at 14 years: $13,296.63 vs $12,917.01 and lower QALY (5.37 vs 6.01) at 14 years in un-resurfaced group. NICE’s translation and interpretation to the UK healthcare system: Patella resurfacing results in £263 saving per patient (2015 US dollar to Pound Stirling currency exchange rates used) un-resurfaced total cost and 0.64 extra QALYs [12].	Patella resurfacing was cost-effective according to the data included in the model.
Murray et al. [22]	Cost utility analysis performed within the UK Knee Arthroplasty Trial (KAT) randomised controlled trial (RCT) Follow-up duration: 10 years Un-resurfaced patella (*n* = 854) Patella resurfacing (*n* = 861)	Un-resurfaced group: 2.8% had late resurfacing in the first 5 years postoperatively. Resurfaced group: 1% had re-operations for complications of the resurfacing during the second 5 years. Findings were independent of trochlear design. Un-resurfaced total costs at 10 years: £8889 Patella resurfacing total costs at 10 years £8,785 Patella resurfacing saves £104 per person. Un-resurfaced vs resurfaced patellae QALY: 5.110 vs 5.297 Patella resurfacing results in trend towards 0.187 increase in QALYs.	Patella resurfacing may be more cost-effective.

Although the results were in favour of both lower rates of secondary surgery and reduced overall total costs for resurfaced patellae in both studies, some issues are worth highlighting [22, 23]. Firstly, Weeks et al. showed that if the secondary resurfacing rate were reduced to <0.5% in the un-resurfaced group of patients there would be no overall difference in costs between resurfacing and not resurfacing according to a one-way sensitivity analysis [23]. Secondly, Murray et al. reported a mean QALY increase of 0.187 in resurfaced patellae with a 95% confidence interval – 0.025 to 0.399; *p* = 0.08. The authors describe this as ‘a non-significant trend towards improved quality of life’ [22].

It is important to balance the costs of complications related to primary patella resurfacing with the costs of managing an un-resurfaced patella when establishing a monetary viewpoint. It is also vital to identify patients who may be unsuitable for primary resurfacing in the decision-making process (for example, those with insufficient bone stock).

### Key points

Overall, patella resurfacing may be more cost-effective, resulting in an increase in QALYs gained. This is consistent with the 2020 NICE recommendations for patella resurfacing [12, 22, 23].

### The influence of implant design on outcomes

TKA component design may influence knee biomechanics and performance. Early designs gave little consideration to the patellofemoral joint and resulted in complications in up to 50% of cases [29].

### Femoral component

The high rates of patellofemoral complications have led to the development of patella-friendly components. Those with a deeper trochlea groove and larger lateral flange have been shown to improve patella tracking and stability [30, 31]. However, a study of 10,157 TKAs (9530 PPR, 627 PPNR) found no significant difference in revision rates between various patella-friendly and earlier designs [15].

### Patella component

Patella components can be cemented or non-cemented, inlayed or onlayed, metal-backed or all-polyethylene, and are available in various morphologies. Inlay and onlay patellae have shown similar kinematic profiles in laboratory studies, but the relative ease of application and potential to medialise to improve tracking has led to a preference for onlay [32, 33]. Registry data shows that onlay cemented patella resurfacings are the most commonly used in the UK and are associated with good outcomes [2]. More important than the prosthesis is the positioning, as asymmetric resection may lead to patella tilt [33, 34], and failure to medialise can result in increased shear forces [35].

### Prosthesis constraint

Registry data suggests that rates of SPR are higher in posterior-stabilised (PS) TKA. Analysis of the New Zealand Joint Registry found PS-TKAs were at increased risk of undergoing SPR with an odds ratio of 1.9 (95% CI 1.34–2.69, *p* < 0.0001) [36]. This is consistent with previous literature [37] and data from the UK NJR which show higher rates of revision for implants with a PS design [2]. The revision rate of the PFC Sigma bicondylar knee cemented PS knee at 10 years was 2.6% (2.36–2.87) and 4.37% (3.99–4.78) for PPR and PPNR respectively. Whereas the Attune PS[Fem] fixed bearing (FB) [tib] revision rate was 2.93% vs 3.85% for PPR and PPNR at 10 years, respectively. PS Gen II Oxinium [Fem] Gen II [tib] 7.09% vs 15.2% at 13 years. PS Gen II [fem and tib] 4.38% vs 4.47% at 13 years (interestingly at 15 years the PPNR group increases to 6.86% but there is no comparative data for the PPR group). In contrast, the rates of cumulative revision are consistent between resurfaced and non-resurfaced TKAs for unconstrained TKAs where the cruciate ligaments are retained (CR).

The mechanism for this discrepancy is not clear: it has been hypothesised that the use of PS-TKA is indicated in more complex cases and pre-existing ligamentous deficiency, therefore biasing outcomes [36].

### Key points

Patella-friendly prostheses are yet to eradicate anterior knee pain. Onlay cemented patella resurfacing is the most popular design and is associated with improved survivorship. Registry data suggest PS TKA may be an additional indication for resurfacing.

### Risks associated with primary patella resurfacing

Proponents of primary patella non-resurfacing (PPNR) may quote risks of complications related to patella resurfacing such as patella fracture, mal-positioning, mal-tracking and tendon rupture [38].

### Patella fracture

Patella fracture is the second most common type of periprosthetic fracture after supracondylar fractures [39]. Patella fractures can be challenging to manage and a systematic review found that 69% of patella fractures are managed conservatively [40]. They can occur in resurfaced and non-resurfaced patellae, although the majority (99%) are associated with resurfacing [40, 41]. The mechanism of injury is most commonly atraumatic or low energy trauma, and the majority are asymptomatic [40]. In addition to the patella resurfacing, risk factors for fracture can be grouped into patient factors (e.g. osteoporosis, male, inflammatory arthropathy, obesity), implant design factors (e.g. cementless implants, large central peg) and operative factors (e.g. patella devascularisation, inappropriate use of patella clamp, component malpositioning or excessive resection) [38]. A systematic review of 23 studies estimated the mean incidence of patella fracture after TKA to be 1.19% (range 0.15 and 12%) [40]. However, the article reporting the highest rate of fracture was a series of 41 patients that underwent isolated patella component revision, a major risk factor for patella fracture [42]. Whereas the incidence of patella fracture in non-resurfaced knees was 0.05% [43]. In a retrospective review of 12,464 TKAs between 1985 and 1998 at an American institution, the patella fracture rate was 0.68% [44]. All fractures were associated with patella resurfacing, but the prevalence of PPR within the cohort was not reported. The latest NJR report calculates 0.17 of revision TKAs were performed for peri-prosthetic fractures per 1000 prosthesis-years [2] although there is no specific estimate for patella fractures. In any case, the absolute incidence of patella fracture was low.

### Aseptic loosening

Historically, rates varied from 0.6 to 4.8%, but aseptic loosening has decreased since metal-backed patella resurfacings, which had high rates of failure, have been abandoned [45]. Risk factors for aseptic loosening include obesity, lateral release and joint elevation. Although aseptic loosening is a predominant mechanism of TKA failure overall [46], cases of patella loosening are rare. In a series of 310 TKAs with PPR performed between 2001 and 2008 and followed up for a mean of 96 (58–144) months, there was one (0.3%) case of aseptic loosening that presented at 62 months [45]. The presence of a patella resurfacing creates another source of polyethylene wear which could contribute to aseptic loosening of the tibial or femoral components, but the latest NJR report shows higher rates of revision for aseptic loosening in cemented TKA (both CR and PS) when the patella was not resurfaced [2].

### Avascular necrosis

Extensive lateral release is often attributed as the mechanism for patella devascularisation and has been shown to increase the risk of aseptic loosening and patella fracture 3.8-fold and 2.7-fold, respectively [47]. In a retrospective study of 844 primary TKAs that underwent revision, avascular necrosis of the patella was the reported indication in only 2 (0.2%) cases [46].

### Patella clunk syndrome

Patella clunk syndrome has been described following both PPR and PPNR [48]. The estimated incidence is 0–20% depending on the design of the prosthetic patella [49]. In a systematic review investigating the aetiology of patella clunk syndrome, 3 of 30 studies identified PPR as a significant risk factor, and 1 of 30 suggested that PPR was a non-significant risk factor, with the remaining making no comment [49]. A recent randomised controlled study investigating patella crepitus, a pathology related to patella clunk, in resurfaced and non-resurfaced posterior-stabilised TKAs found the incidence was lower in the PPR group (7.3% vs 23.1%) [50]. Avoiding known risk factors for patella clunk such as smaller patella button size and decreased composite patella thickness [51] may reduce the incidence further.

### Overstuffing

Overstuffing of the patellofemoral compartment is influenced by the femoral component size and position as well as patella thickness. Overstuffing risks higher joint reaction forces, patella fracture, reduced range of motion and anterior knee pain; understuffing risks a reduction in knee extension strength, extensor lag and instability [52]. Despite these theoretical risks, recent clinical studies correlating radiographic appearance and clinical outcomes have been mixed [53–55].

### Instability

Patient, implant and operative factors all contribute to patella instability [38]. Specific considerations when resurfacing the patella are to medialise the patella button to mitigate the need for lateral retinacular release [56], but not so excessively as to cause lateral patella tilt. Most cases of instability are related to internal rotation of the femoral or tibial component therefore not limited to PPR [57, 58]. Historic incidence of patella instability is between 1 and 20% [59]; however, in a retrospective analysis of 255 posterior stabilised-TKAs with PPR, there were no cases of patella instability and 5 cases of mild lateral patella tilt [60].

### Extensor mechanism disruption

Extensor mechanism failure via damage to the quadriceps tendon, patella or patella tendon is a potentially catastrophic complication with high morbidity [61]. Quadriceps tendon injury is rare, occurring in 0.1% of TKAs [61]. Patella tendon injury is reputedly the most difficult extensor mechanism injury to treat, with complication rates of 63% [62] and an incidence of 0.17% [63]. Patella fracture is the most common extensor mechanism injury after TKA and 99% are associated with PPR but the majority do not result in a disruption [44] (see above evidence for patella fractures). Risk factors, both intra- and post-operatively, are numerous and are usually linked to direct trauma at the time of surgery or suboptimal implant positioning leading to increased stress when ambulating. Extensor mechanism failure is rare, accounting for 1.2% of revision TKAs [46]. In a series of 860 PPR TKAs, 1.3% sustained patella fractures and 0.47% sustained patella tendon rupture, although the authors highlighted that most fractures were secondary to trauma and patella tendon ruptures followed previous surgery such as valgus osteotomy [64].

### Key points

Despite the potential pitfalls of patella resurfacing described in the literature, the absolute risks are low and continue to decrease with improving implant designs [65]. With diligent patient selection, pre-operative planning and attention to intraoperative technique the risks can be reduced further [52] (Table 3).

**Table 3:** Techniques and considerations to mitigate the risk of complications following PPR

Complication	Consideration/technique
Patella fracture	Using all-polyethylene patella button Avoiding button with large central peg Avoiding excessive patella resection
Patella avascular necrosis	Avoiding excessive lateral retinacular release Avoiding excessive patella reaming
Patella clunk	Femoral component: low intercondylar box ratio Avoiding significant changes in joint line height, reduced patella thickness, using a small patella button Maintaining posterior condylar offset
Under/overstuffing	Restoring native patella thickness Using appropriate size femoral component without anteriorisation
Patella instability	Using femoral component with extended and deep trochlea groove Attention to internal rotation of femoral component Tibial component external rotation and centralisation Medialising and superiorisation of patella button Performing a lateral patella facetectomy if necessary
Extensor mechanism disruption	Avoiding aggressive eversion of patella Avoid change of joint line height

### The fate of non-resurfaced patellae

Surgeons opting not to resurface the patella may be reassured by the appearance of healthy patella cartilage intra-operatively. However, an RCT of 125 TKAs assessed the patella cartilage on a scale of 0 (pristine cartilage) to 12 (eburnated) at time of surgery and although lower scores correlated with better outcomes, there were cases of high scores with good outcomes and vice versa, highlighting the unpredictable nature of selective resurfacing [11]. Furthermore, those that returned for SPR were evenly distributed between high and low patella scores.

#### Cellular changes of patella cartilage when everted during surgery

During TKA, the patella is often everted to gain access. In a rabbit model, exposure of hyaline cartilage to air for more than 60 min caused full thickness chondrocyte necrosis, although, when reviewed at 3 weeks, there was no evidence of necrosis, hence chondrocyte necrosis may be potentially reversible [66]. Laminar flow was associated with increased rate of chondrocyte necrosis versus static air, and although rehydration macroscopically restores cartilage, there was no reversal of chondrocyte necrosis. These deleterious effects were prevented using a saline soaked gauze [67].

#### Interaction of metal-on-cartilage for PPNR

Cartilage is damaged following interaction with metal implants [68], with decreased cell viability, extracellular matrix disruption and increased wear when compared to cartilage-on-cartilage interaction [69]. This unfavourable interaction may partially explain the higher rates of anterior knee pain seen with PPNR. Over time, the native patella may become eroded. In a RCT involving 125 TKAs, all 6 non-resurfaced knees that returned for SPR had developed radiological evidence of wear and half were found to have bone loss intra-operatively [11].

### Key points

The fate of the non-resurfaced patella is not evidenced but is unlikely to flourish in a prosthetic environment after surgery.

## Conclusion

The authors suggest routine PPR in line with NICE guidelines, based on evidence of at least equivocal clinical outcomes, lower re-operation rates, economic viability and low risk of complications with modern implants and sound surgical technique. Adequately powered randomised controlled trials with appropriate outcome measures and sufficiently long follow-up are needed to give a definitive answer to this clinically relevant issue.

## Abbreviations

AJRR: American Joint Replacement Registry
AKP: Anterior knee pain
CI: Confidence intervals
CR: Cruciate retaining
fem: Femoral component
FB: Fixed bearing
KAT: Knee Arthroplasty Trial
KSS: Knee Society Score
NICE: National Institute for Health and Clinical Excellence
PROM: Patient Reported Outcome Measure
PFJ: Patellofemoral joint
PPNR: Primary patella non-resurfacing
PPR: Primary patella resurfacing
PFC: Press-fit condylar
PS: Posterior-stabilised
QALY: Quality adjusted life years
RCT: Randomised controlled trial
SPR: Secondary patella resurfacing
TKA: Total knee arthroplasty
Tib: Tibial component
UK NJR: United Kingdom, National Joint Registry
